# Multi-Class Classification of Breast Cancer Subtypes Using ResNet Architectures on Histopathological Images

**DOI:** 10.3390/jimaging11080284

**Published:** 2025-08-21

**Authors:** Akshat Desai, Rakeshkumar Mahto

**Affiliations:** 1 Department of Computer Science, California State University, Fullerton, CA 92831, USA; akshatdesai@csu.fullerton.edu; 2 Department of Electrical and Computer Engineering, California State University, Fullerton, CA 92831, USA

**Keywords:** deep learning, convolutional neural networks (CNNs), ResNet architecture, breast cancer

## Abstract

Breast cancer is a significant cause of cancer-related mortality among women around the globe, underscoring the need for early and accurate diagnosis. Typically, histopathological analysis of biopsy slides is utilized for tumor classification. However, it is labor-intensive, subjective, and often affected by inter-observer variability. Therefore, this study explores a deep learning-based, multi-class classification framework for distinguishing breast cancer subtypes using convolutional neural networks (CNNs). Unlike previous work using the popular BreaKHis dataset, where binary classification models were applied, in this work, we differentiate eight histopathological subtypes: four benign (adenosis, fibroadenoma, phyllodes tumor, and tubular adenoma) and four malignant (ductal carcinoma, lobular carcinoma, mucinous carcinoma, and papillary carcinoma). This work leverages transfer learning with ImageNet-pretrained ResNet architectures (ResNet-18, ResNet-34, and ResNet-50) and extensive data augmentation to enhance classification accuracy and robustness across magnifications. Among the ResNet models, ResNet-50 achieved the best performance, attaining a maximum accuracy of 92.42%, an AUC-ROC of 99.86%, and an average specificity of 98.61%. These findings validate the combined effectiveness of CNNs and transfer learning in capturing fine-grained histopathological features required for accurate breast cancer subtype classification.

## 1. Introduction

Breast cancer is one of the leading causes of mortality among women across the globe. According to data reported by the WHO in 2022, approximately 2.3 million women around the world were diagnosed with breast cancer, among whom 670,000 lost their lives [1]. One of the major causes behind such a high number is late-stage diagnosis, which means the disease has already progressed to an advanced stage where treatment options are limited. Therefore, early detection is critical to confirm the presence of cancer and determine its specific subtype since the prognosis, treatment response, and progression depend on subtype classification. Hence, there is an urgent need to develop an automated, efficient, cost-effective, and objective diagnostic tool to identify the breast cancer subtype. This is crucial since it enables physicians to provide an effective treatment plan, ultimately improving the patient’s survival rate.

Traditionally, breast cancer is diagnosed through histopathological examination [2,3,4,5], where tissue samples are analyzed by pathologists under a microscope and classified as either benign or malignant. Similarly, other common diagnostic techniques employed include mammography [6,7,8], ultrasound [9,10,11], magnetic resonance imaging (MRI) [12], and biopsy-based histopathology [13,14]. All of these techniques are effective in diagnosing breast cancer. However, each has certain shortcomings. Mammography-based breast cancer screening can result in false positives or negatives for women with dense breast tissue. Ultrasound- and MRI-based diagnostic techniques are very effective; however, their dependence on a skilled operator has made them expensive and inaccessible in resource-limited settings. Although histopathology is considered the gold standard in breast cancer diagnosis, it is labor-intensive and prone to inter-observer variability, which can result in inconsistent diagnoses among pathologists, particularly in high-volume diagnostic workflows. Given the challenges associated with each of these techniques, it is crucial to have a reliable, cost-effective solution that can assist medical professionals, reduce diagnostic workload, and enhance accessibility. This can be achieved with the help of artificial intelligence (AI) and machine learning (ML). By leveraging these advanced techniques together with deep learning on histopathological images, human dependence on diagnosing or classifying cancer types can be reduced while improving diagnostic accuracy. While AI cannot replace the need for invasive biopsy procedures or specialist oversight, it serves as an efficient decision-support system that has the potential to enhance accuracy and consistency in breast cancer subtype classification.

Many studies have applied deep learning models to classify breast cancer using histopathological images, mammograms, and ultrasound scans. For example, convolutional neural networks (CNNs) are widely used in binary classification to distinguish between benign and malignant tumors. In [15], the authors used deep learning-based computer-aided diagnosis (CAD) systems to classify mammographic mass lesions, significantly enhancing diagnostic accuracy to 98.94% and reducing reliance on unnecessary biopsies. Similarly, in another study, CNN-based classification was applied to the mini-MIAS database, consisting of mammographic images, achieving an impressive accuracy of 89.05% and a sensitivity of 90.63% [16]. Transfer learning is another technique where pretrained deep learning architectures, such as VGG16, Inception, and ResNet, have shown remarkable accuracy in automating breast cancer detection in mammographic and histopathological images [17,18,19]. For example, Saber et al. [17] showed that through transfer learning with models such as VGG16 and ResNet-50, breast cancer can be diagnosed using mammographic images, with an impressive accuracy of 98.96%. Another study by Shahidi et al. [19] showed that using preprocessing, data augmentation, and model selection techniques, transfer learning models like ResNeXt and SENet can improve breast cancer classification.

Although advancements using deep learning models have led to significant progress in breast cancer diagnosis, most existing studies mainly focus on binary classification (benign vs. malignant) rather than on differentiating specific histopathological subtypes. Breast cancer is a heterogeneous disease; hence, it has multiple subtypes, each of which requires its own treatment. However, researchers have conducted limited work with deep learning models for multi-class classification of breast cancer subtypes using histopathological images. Due to this research gap, it is essential to study the ability of CNN-based deep learning architectures such as ResNet to classify the various histopathological subtypes of breast cancer. Hence, in this work, we examine various ResNet architectures for multi-class classification of breast cancer subtypes using histopathological images. Moreover, in this work, we employ the ResNet-18, ResNet-34, and ResNet-50 models to evaluate their performance in distinguishing between eight tumor subtypes in the BreaKHis dataset [20] at multiple magnifications (40X, 100X, 200X, and 400X). By analyzing the effectiveness of these deep learning models, this study will advance current efforts to automate histopathological classification, which will eventually reduce diagnostic subjectivity and improve the accuracy of breast cancer subtype identification.

The remainder of this paper is organized as follows. Section 2 discusses relevant studies conducted using the BreaKHis dataset. Section 3 presents the methodology utilized in this work to examine the performance of the various ResNet models. Section 4 presents the results, where various metrics are used to evaluate the performance of the different ResNet models. Then, we conclude by describing the implications of this study and its effects on breast cancer diagnosis, as well as potential directions for future research.

## 2. Related Works

In this study, we utilize the BreaKHis dataset, which has been widely used as a benchmark for testing AI-based models in breast cancer diagnosis [20]. The dataset consists of 7909 histopathological images from 82 patients, which are categorized into benign and malignant tumors. Each category is further divided into four subtypes. Additionally, the dataset provides images at four different magnification levels, including 40X, 100X, 200X, and 400X, meaning deep learning models are trained on varying image resolutions. Researchers have conducted various studies using the BreaKHis dataset to apply ML and AI models for diagnosing breast cancer. Some of the key techniques are outlined below.

### 2.1. Machine Learning Techniques

Initial research using the BreaKHis dataset for diagnosing breast cancer included utilizing traditional machine learning techniques to classify histopathological images. For this purpose, handcrafted features were first extracted, followed by applying classifiers such as support vector machine (SVM), k-nearest neighbor (k-NN), decision tree, and random forest. For instance, in [21], Alqudah et al. utilized sliding-window-based feature extraction using Local Binary Pattern (LBP) features, where they divided each image into 25 sliding windows for localized feature extraction. These extracted features were then utilized to train a support vector machine (SVM) classifier, achieving an impressive accuracy of 91.2%. Another study by Ariateja et al. extracted features that included color, Gabor filter, and GLCM descriptors, which were later utilized to train a weighted k-nearest neighbor (weighted k-NN) algorithm to classify histopathological images [22]. The method proposed in [22] achieved classification accuracies of 90% at 40X, 100X, and 200X magnifications, and 89.6% at 400X, demonstrating its potential for supporting breast cancer histopathology analysis. In a similar study by Murtaza et al. [23], the authors trained a decision tree model using the BreaKHis dataset and fine-tuned it on the Bioimaging Challenge 2015 dataset. Using a misclassification reduction algorithm, they achieved a classification accuracy ranging from 87.5% to 100% across four breast tumor subtypes. All these traditional machine learning techniques have shown promise. However, they have limitations due to their overreliance on feature extraction. Hence, conventional machine learning techniques are unable to capture complex patterns inherent in histopathological images.

### 2.2. Deep Learning Models for Binary Classification

Similarly, earlier work on applying deep learning models to the BreaKHis dataset focused primarily on binary classification using CNNs. These models focused only on differentiating between benign and malignant tumors. Araújo et al. in [24] trained a CNN model using the BreaKHis dataset, achieving an accuracy of 83.3% across all magnifications. Similarly, in another work by Spanhol et al., a patch-based CNN model was utilized for feature extraction and classification, achieving an accuracy of 85.6% at 200X magnification [20]. These works underscore the potential of deep learning models to automatically learn hierarchical features from histopathological images.

### 2.3. Transfer Learning and Model Optimization

The use of deep learning for the BreaKHis dataset was further advanced by Bayramoglu et al. through the combined use of CNNs with transfer learning, resulting in accuracy improving to 87.3% at 400X magnification [25].

A further advancement in breast cancer classification for benign and malignant tumors was achieved through a hybrid of a CNN and Long Short-Term Memory (LSTM) with federated learning, resulting in an accuracy of 93% [26]. A similar hybrid technique was utilized by Kaddes et al. in [27], achieving an impressive accuracy of 99.90%. Besides hybrid techniques, various deep learning models, including ResNeXt-50, DPN131, and DenseNet-169, have been utilized to classify binary cancer types, achieving an impressive accuracy of 99.5% [28]. This shows the impact and progress made in distinguishing benign from malignant tumors due to advancements in deep learning.

However, even when AI demonstrates its potential in breast cancer diagnostics through binary classification, it is not sufficient to capture the full heterogeneity of breast cancer [29]. Since breast cancer consists of multiple histopathological subtypes, developing a multi-class classification model is essential to ensure that patients can receive the proper treatment plan.

### 2.4. Deep Learning Models for Multi-Class Classification

Although binary classification in breast cancer research using deep learning shows great promise and potential, it is not sufficient, since subtype classification is essential for personalized treatment. For this purpose, multi-class classification of histopathological subtypes of breast cancer is necessary. In the BreaKHis dataset, there are eight classes into which images are categorized: adenosis (A), ductal carcinoma (DC), fibroadenoma (F), lobular carcinoma (LC), mucinous carcinoma (MC), papillary carcinoma (PC), phyllodes tumor (PT), and tubular adenoma (TA). Research was conducted in this direction by Umer et al., who proposed a six-branch deep convolutional neural network (6B-Net) with feature fusion and selection mechanisms for multi-class breast cancer classification [30]. When this model was applied to the BreaKHis dataset to classify histopathological images into eight breast cancer classes, an accuracy of 90.10% was achieved. Another research work adopted a DenseNet121-based deep learning model that achieved an average accuracy of 92.50% [31].

The BreaKHis dataset enables advancements in AI-driven breast cancer diagnosis, which can result in significant progress in binary and multi-class classification tasks. Traditional machine learning models, although not very accurate, provide the groundwork for integrating advanced deep learning models to further improve classification accuracy. Although the use of deep learning, transfer learning, and model optimization has significantly improved the accuracy of binary classification, much work still needs to be done in multi-class classification. The complexity and heterogeneity of breast cancer make further advancements in multi-class classification necessary to enable precise and personalized treatment planning.

## 3. Dataset and Preprocessing

### 3.1. BreaKHis Dataset

**Figure 1:**
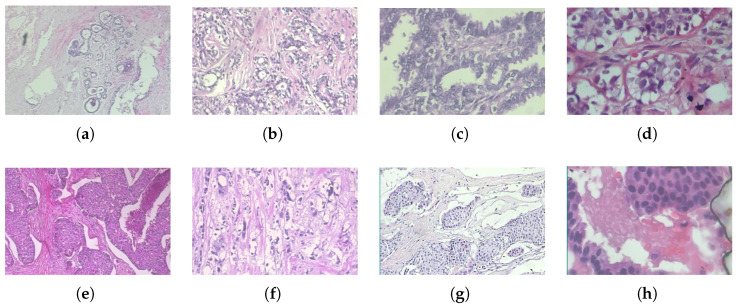
Histopathological images of different breast tumor subtypes from the BreaKHis dataset at various magnifications: (**a**) adenosis (40X), (**b**) fibroadenoma (100X), (**c**) phyllodes tumor (200X), (**d**) tubular adenoma (400X), (**e**) ductal carcinoma (40X), (**f**) lobular carcinoma (100X), (**g**) mucinous carcinoma (200X), and (**h**) papillary carcinoma (400X).

The BreaKHis dataset is publicly available [20] and consists of histopathological breast cancer images widely used for benchmarking machine learning and deep learning models for breast cancer classification. The dataset contains 7909 microscopic images obtained from 82 patients. Each image in the dataset corresponds to a breast tumor specimen extracted through biopsy procedures, as shown in Figure 1. Additionally, each image is available at four different magnification levels (40X, 100X, 200X, and 400X), capturing tissue structures at varying resolutions and enabling multi-scale feature learning, as shown in Table 1. The lower magnifications (40X, 100X) are ideal for broader tissue morphology, whereas the higher magnifications (200X, 400X) show detailed cellular structures, which are crucial for deep learning models in differentiating tumor subtypes.

Each tumor sample in the dataset is further classified into four subcategories for both benign and malignant cases, as shown in Table 2. In oncology, precise information regarding the varied growth patterns, aggressiveness, and treatment responses of different cancer types is vital, which can only be achieved through multi-class classification. Given that the dataset is imbalanced, with malignant cases significantly outnumbering benign cases, data processing and augmentation are essential to train and build a robust model.

### 3.2. Preprocessing and Augmentation

For applying deep learning models for image classification of histopathological data, it is essential for the model to effectively learn discriminative features while mitigating variations in staining techniques, imaging conditions, and tissue structures. A robust preprocessing pipeline is especially necessary for the BreaKHis dataset since the images in this dataset are of varying resolutions, intensity distributions, and orientations.

#### 3.2.1. Image Resizing

As discussed earlier, the BreaKHis dataset consists of images at different magnification levels (40X, 100X, 200X, and 400X), leading to variations in spatial resolution. Deep learning models such as CNNs require input images to be of uniform size for batch processing. Hence, to ensure uniformity and compatibility with pretrained architectures such as ResNet, all images were resized to a standard resolution of 224 × 224 pixels. The resizing operation maintains spatial consistency across different magnifications, reduces computational overhead, and ensures compatibility with ImageNet-pretrained models. Although image resizing causes a loss of fine-grained cellular details, the use of deep feature extraction layers in the ResNet architecture compensates for this loss by capturing hierarchical spatial information.

#### 3.2.2. Normalization

The next step in preparing the dataset for training and testing was normalization. This step is crucial for deep learning since it standardizes image intensity distributions, stabilizing training and improving model convergence. Among the various techniques, in this work, mean-variance normalization was applied to scale pixel values to a zero-mean, unit-variance distribution:

(1) $$I^{\prime }=\frac{I-\mu }{\sigma }$$

where $I^{\prime }$ is the normalized image, *I* represents the original pixel intensity, μ is the dataset-wide mean, and σ is the standard deviation. This transformation reduces the variability introduced by staining differences in histopathological slides and ensures a consistent input distribution.

### 3.3. Data Splitting Strategy

To train the ResNet models and later evaluate them, we randomly split the dataset into three subsets that included 80% for training, 10% for validation, and the remaining 10% for testing. The use of a random split ensures that each subset contains a diverse representation of the eight breast cancer subtypes, ensuring that the trained model can generalize well across unseen samples. In this work, stratified sampling was not used due to the multi-class nature of the classification task; instead, random shuffling was performed prior to splitting to ensure variability across sets.

### 3.4. Data Augmentation

Data augmentation is applied when a dataset contains a limited number of images. Hence, using this technique, the training dataset was expanded artificially by applying a series of transformations to improve the model’s robustness and generalization. This technique is appropriate since the BreaKHis dataset contains limited histopathological images. The data augmentation process introduces variations in image orientation and appearance while preserving the essential structural patterns required for classification, reducing the possibility of overfitting. In this work, augmentation was applied through the PyTorch (version 2.5.1+cu124) transforms pipeline.

#### 3.4.1. Random Horizontal Flipping

Histopathological slides can exhibit variations in tissue orientation due to the preparation process. Hence, to prevent potential biases arising from these positional differences, random orientation flipping is employed. In this work, random flipping of images with a probability of 50% was applied. This ensures that the model is not biased toward a particular tissue orientation.

#### 3.4.2. Random Rotation ($\pm 10^{\circ }$)

Random rotation of images within the range of ±10° was applied to account for the possible variation in slide position under the microscope.

(2) $$\left[\begin{array}{c} x^{\prime }\\ y^{\prime } \end{array}\right]=\left[\begin{array}{cc} \cos\theta & -\sin\theta \\ \sin\theta & \cos\theta \end{array}\right]\left[\begin{array}{c} x\\ y \end{array}\right]$$

where (*x*, *y*) represent the original pixel coordinates, ($x^{\prime }$, $y^{\prime }$) represent the rotated coordinates after transformation, and θ is the randomly selected rotation angle between −10° and +10°.

### 3.5. Handling Class Imbalance

In the BreaKHis dataset, malignant tumor samples significantly outnumber benign ones, which can result in the deep learning model being biased toward the majority class. Hence, in this work, instead of applying popular class balancing techniques such as oversampling, undersampling, or weighted loss functions, we applied random shuffling.

## 4. Methodology

This study proposes an automated deep learning-based classification framework leveraging Residual Networks (ResNet-18, ResNet-34, and ResNet-50) for classifying eight distinct tumor subtypes. The model architecture is designed to efficiently extract and learn hierarchical feature representations, enabling multi-class classification with high accuracy. The methodology employed in this study consists of multiple stages, including data acquisition, model architecture, training strategy, and evaluation.

### 4.1. Overview of the Proposed Model

**Figure 2:**
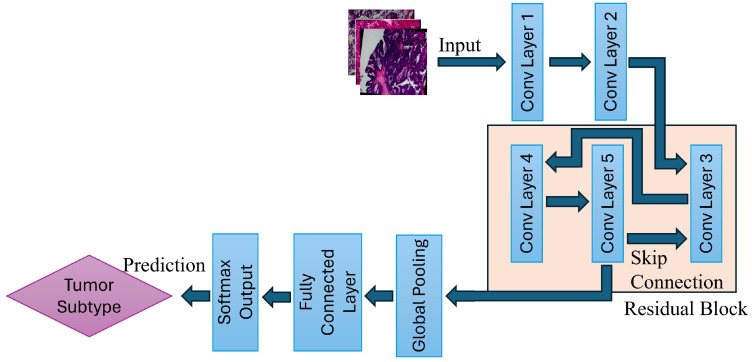
Illustration of the ResNet-based multi-class breast tumor classification model. The pipeline consists of convolutional feature extraction, residual block learning, global pooling, and final classification via a fully connected layer.

The proposed deep learning framework follows a structured pipeline that begins with the acquisition of images from the BreaKHis dataset, followed by preprocessing and augmentation, including image resizing, normalization, random horizontal flipping, and random rotation. Then, the processed dataset is fed into the ResNet-based CNN architecture for classification. The ResNet model employed in this work then performs convolutional feature extraction and residual learning, and uses fully connected classification layers, which allow for accurate differentiation among eight breast tumor subtypes, as shown in Figure 2.

### 4.2. Deep Residual Network (ResNet) for Tumor Classification

By addressing the vanishing gradient problem, ResNet has revolutionized deep learning applications in image classification. Traditional CNN models are effective techniques for various applications; however, as the architecture grows in depth, the signal that guides the network’s learning process dwindles to near insignificance during backpropagation. This makes the learning process ineffective, eventually making the network incapable of effectively refining its parameters. ResNet rectifies this issue through the use of a skip connection, as shown in Figure 2.

The output of a residual block is computed as

(3) H(x)=F(x,W)+x

where *x* is the input to the residual block, F(x,W) is the residual function learned by the network, and *W* represents the weight matrices of the convolutional layers.

This can be expanded further into a two-layer residual block. The transformation is defined as

(4) $$y=\mathrm{ReLU}\left(W_{2}\cdot \mathrm{ReLU}\left(W_{1}\cdot x+b_{1}\right)+b_{2}\right)+x$$

where $W_{1}$ and $W_{2}$ are the convolutional weight matrices, and $b_{1}$ and $b_{2}$ are the bias terms.

### 4.3. ResNet Architecture

This work implemented three variations of ResNet, including ResNet-18, ResNet-34, and ResNet-50, to evaluate the effectiveness of network depth in classifying the various breast cancer classes. Each of these architectures differs in terms of the number of layers and computational efficiency, as shown in Table 3. ResNet-18 and ResNet-34 use a basic residual block consisting of two 3 × 3 convolutional layers, whereas ResNet-50 incorporates bottleneck residual blocks, where each block consists of three convolutional layers (1 × 1, 3 × 3, 1 × 1 convolutions). The bottleneck residual block reduces the number of computations while maintaining superior feature extraction.

### 4.4. Computational Setup and Training Time

ResNet training and testing were conducted on a high-performance workstation. This workstation is equipped with an NVIDIA A100 GPU (40 GB of VRAM) (NVIDIA Corporation, Santa Clara, CA, USA), an 8-core CPU, and 32 GB of RAM, and was provided through the National Research Platform (NRP) program [32]. All the histopathological images were locally stored on the workstation where the Python (version 3.12.8) scripts were executed. Simultaneously, the performance of the three ResNet architectures was measured. Among the three selected ResNet models, ResNet-50 took around 30 min to an hour to train over 20 epochs.

### 4.5. Model Training Process

The training process of the ResNet deep learning model follows a structured pipeline, which ensures efficient feature extraction and residual learning ideal for multi-class breast tumor subtype identification. The training mechanism involves mini-batch processing, backpropagation, and optimization using the Adam optimizer.

Each image first undergoes preprocessing and augmentation, after which it is fed into the ResNet models. All images then pass through multiple convolution processes, where hierarchical features from the images are extracted. The early stage captures low-level features such as edges and textures, while deeper layers learn high-level tumor structures. Then comes the residual learning mechanism in ResNet, which is critical for stable gradient propagation through skip connections and for mitigating the vanishing gradient problem, as shown in Figure 2.

#### 4.5.1. Forward Propagation

Forward propagation involves the following processes:

1. Input Image Processing:

Each image undergoes normalization and resizing prior to entering the neural network.

2. Convolutional Feature Extraction:

As shown in Figure 2, the first two convolutional layers extract spatial features such as edges, textures, and cell morphology. These are computed using

(5) $$Y\left(i,j\right)=\sum_{m}\sum_{n}I\left(i-m,j-n\right)\cdot K\left(m,n\right)$$

where Y(i,j) is the input feature map, I(i−m,j−n) represents the input image pixels, and K(m,n) is the convolution kernel.

3. Residual Learning via Skip Connections:

The residual block allows the gradient to flow smoothly and efficiently through the network, which eventually helps prevent the vanishing gradient problem. This is achieved by adding skip connections that bypass one or more layers, enabling the network to learn identity mappings. The skip connection representations are shown in Equation (3) and Equation (4), respectively.

#### 4.5.2. ResNet Model Complexity and Efficiency Comparison

Understanding the computational trade-off between the three ResNet architectures is vital since it allows for evaluating the effectiveness of each model in resource-constrained clinical environments or real-time diagnostic workflows. Beyond classification accuracy, factors such as model size, computational overhead (GFLOPs), and inference latency must be carefully considered before determining the most effective and practical architecture for deployment. The architectural characteristics of the three ResNet variants, ResNet-18, ResNet-34, and ResNet-50, are shown in Table 4. Among the three ResNet architectures, ResNet-18 has a shallow architecture comprising 11.18 million parameters and is the lightest-weight model with the smallest model size of 42.65 MB and inference time of 3.67 milliseconds per image. On the other hand, ResNet-34 has double the number of layers compared to ResNet-18. Finally, ResNet-50 is the deepest among the three selected ResNet models, with 23.52 million parameters and a model size of 89.74 MB. All models process input images resized to 224 × 224 pixels and are configured to classify eight distinct breast cancer subtypes. Transfer learning from ImageNet-pretrained weights was employed to enhance feature extraction capabilities. This comparative analysis will enable assessing the trade-off between architectural complexity and accuracy.

## 5. Results and Analysis

In this section, we evaluate three deep learning models, ResNet-18, ResNet-34, and ResNet-50, to determine their performance in classifying breast cancer subtypes. We utilize multiple performance metrics, such as accuracy, precision, recall, and F1-score, for a well-rounded assessment. Additionally, graphical tools such as loss curves, confusion matrices, ROC curves, and precision–recall (PR) curves are employed to provide comprehensive insights into the models’ training dynamics, predictive behavior, and class-wise performance across multiple evaluation dimensions. Additionally, the effects of various data-balancing techniques and magnifications on accuracy and classification performance are evaluated.

### 5.1. Model Training Dynamics and Convergence

**Figure 3:**
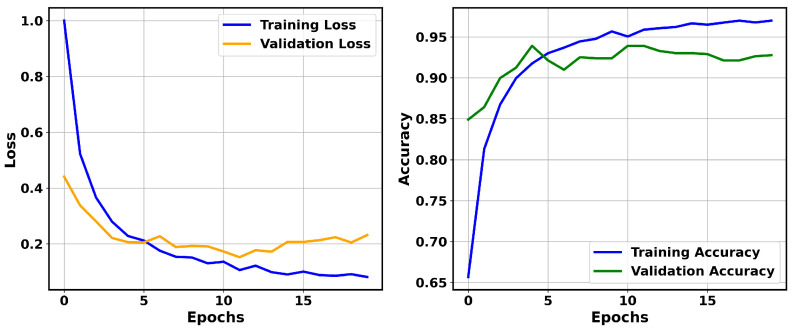
Training and validation loss (**left**) and training and validation accuracy (**right**) over 20 epochs for ResNet-50 on the BreaKHis dataset.

Among the ResNet models utilized in this study, ResNet-50, when run over 20 epochs, achieved superior performance. The validation accuracy of ResNet-50 improved from 84.90% to a peak of 93.91% at epoch 5, after which it oscillated within the range of 92.64% to 93.91%, suggesting stable generalization to unseen data. Similarly, its training accuracy showed a consistent increase, improving from an initial 65.66% to 96.99% by the final epoch. In contrast, its validation loss steadily declined from 0.9998 to 0.0812, as shown in Figure 3. Except for brief fluctuations around epochs 4 and 6, ResNet-50 exhibited a continuous increase in accuracy. Overall, the accuracy and validation loss show that the model effectively learned to discriminate features from the training data while avoiding overfitting.

It is important to evaluate the performance of each of the ResNet models in terms of variability and potential overfitting. Hence, the ResNet-18, -34, and -50 models were evaluated over five random seeds. Given that the BreaKHis dataset is imbalanced, different training, validation, and test sets were used to ensure that the results were not biased by a specific split during each run. The test accuracies for each of the seeds, along with their mean values and 95% confidence intervals, are shown in Table 5. Among the three architectures, ResNet-50 achieved the highest mean test accuracy of 92.42%, with a narrow confidence interval (91.13–93.72%). This shows that, compared to ResNet-18 and ResNet-34, ResNet-50 offers strong consistency and generalization. Similarly, ResNet-18 and ResNet-34 also achieved respectable mean accuracies of 88.74% and 88.46%, respectively.

### 5.2. Confusion Matrix Analysis

**Figure 4:**
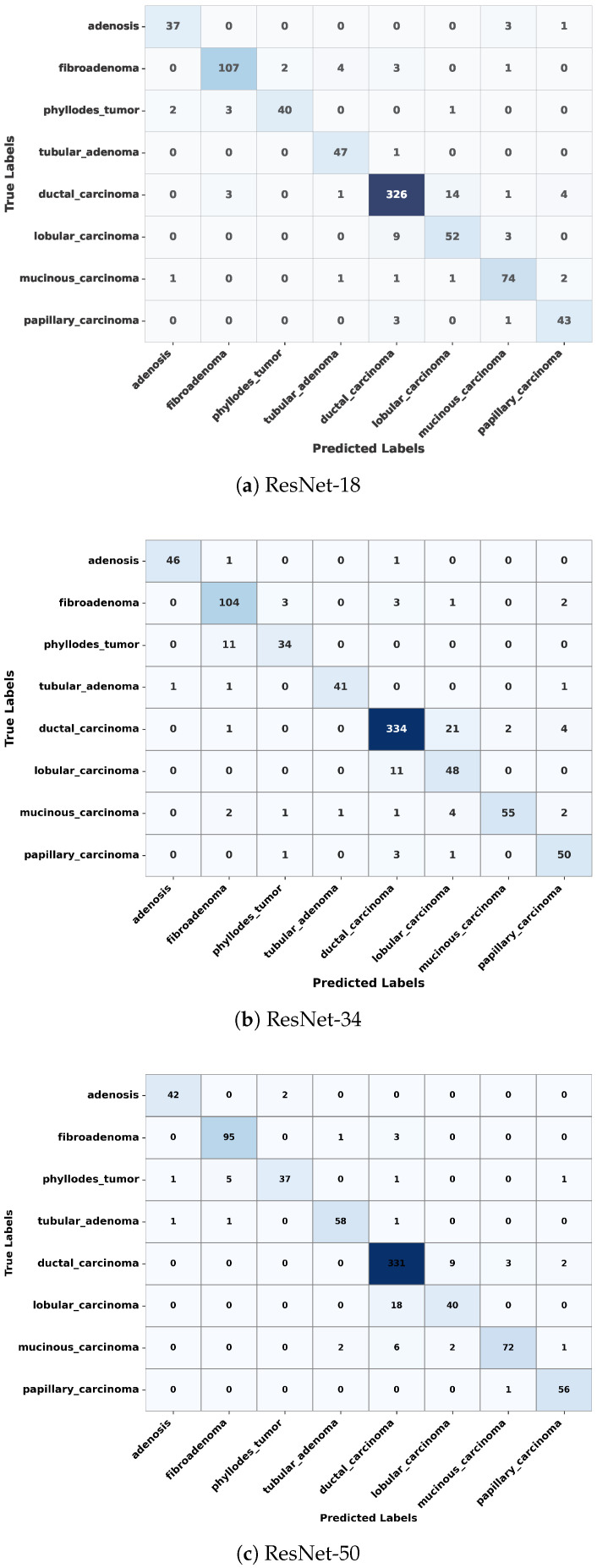
Confusion matrices of ResNet-18, ResNet-34, and ResNet-50 on the test dataset, showing classification performance across eight breast tumor subtypes. Each matrix illustrates correct predictions on the diagonal and misclassifications on the off-diagonal.

In this section, we examine the strengths and residual weaknesses of the trained ResNet models in prediction ability on the test set across subtypes using confusion matrices. The confusion matrices for ResNet-18, ResNet-34, and ResNet-50 are shown in Figure 4. The diagonal dominance indicates high accuracy across most of the classes. The three confusion matrices shown in Figure 4 indicate that the three ResNet models were able to achieve exceptional performance in classifying ductal carcinoma, adenosis, fibroadenoma, and tubular adenoma. However, the models still showed confusion between some of the classes, such as lobular carcinoma and mucinous carcinoma, as well as phyllodes tumor and fibroadenoma, as shown in Figure 4. These misclassifications might have resulted from inherent visual similarities in tissue architecture patterns and morphology among these subtypes. Such confusion can be minimized through the use of a balanced dataset that adequately represents each of the subtypes, thus allowing the model to learn distinctive and discriminative features.

### 5.3. Analysis of Receiver Operating Characteristic (ROC) and Precision–Recall (PR) Curves

**Figure 5:**
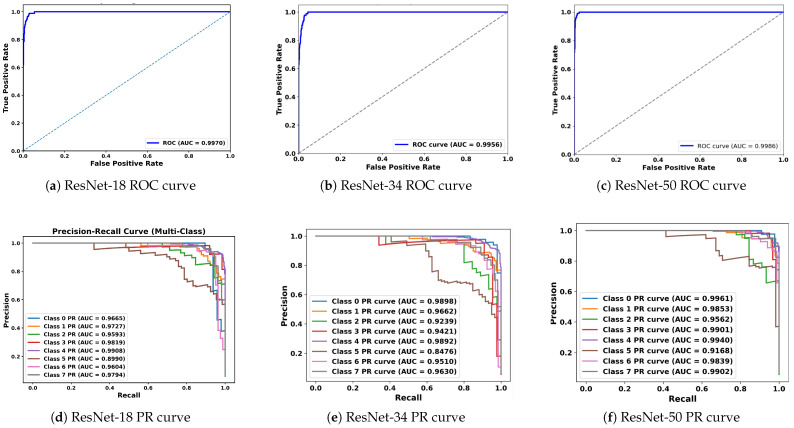
ROC (top) and precision–recall (bottom) curves for ResNet-18, ResNet-34, and ResNet-50 on the test set. ROC panels show the trade-off between sensitivity and specificity across thresholds. PR panels summarize performance under class imbalance by emphasizing precision–recall behavior.

In medical image classification tasks, especially for diseases like cancer, it is essential not only to achieve high overall accuracy but also to rigorously evaluate how well the model distinguishes between different classes. It becomes even more crucial when the dataset is imbalanced or when the cost of false positives and false negatives varies significantly. The ROC curve serves this purpose as it plots the true positive rate (sensitivity) against the false positive rate (specificity) at various decision thresholds. The primary metric derived from the ROC curve is the area under the curve (AUC), which summarizes the model’s ability to correctly classify positive and negative instances across all thresholds. An AUC score of 1.0 means perfect classification, whereas a value of 0.5 implies no discriminative power, which is equivalent to random guessing. In this study, the ROC curves for ResNet-18, ResNet-34, and ResNet-50 demonstrate that each model maintains high true positives while keeping false positives low across various decision thresholds, as shown in Figure 5. The AUC for ResNet-50 was 0.9979, signifying near-perfect classification performance across the eight breast tumor classes. Similarly, for ResNet-34, the AUC was 0.995, indicating exceptional discriminative performance as well. These high AUC values for both ResNet models highlight the capabilities of each in separating tumor classes with minimal overlap in their predictive probabilities.

Although the ROC curve provides valuable insights, it can sometimes give an overly optimistic view in imbalanced datasets, which can result in favoring the dominant classes and reducing performance for the minority classes. In such cases, the precision–recall (PR) curve becomes a more appropriate tool. Typically, in a PR curve, precision (positive predictive value) is plotted against recall (sensitivity), explicitly focusing on the performance for the positive class while ignoring the true negatives, which dominate in imbalanced scenarios. The summary metric that is utilized for the PR curve is the area under the precision–recall curve (PR-AUC). Hence, the PR-AUC measures the model’s ability to correctly identify positive instances while minimizing false positives. This becomes crucial in cases where detecting rare classes has significant consequences. The PR curves for ResNet-34 and ResNet-50, across each of the 8 tumor classes, are shown in Figure 5. ResNet-34 shows strong PR-AUC values for all tumor subtypes, ranging from 0.85 to 0.9898. The lowest PR-AUC is observed for class 5 (lobular carcinoma), either due to lower representation or high similarity with other classes. Similarly, for ResNet-50, the PR-AUC is even more robust, with AUC values above 0.91 and many of the classes exceeding 0.99. This shows that ResNet-50 consistently maintains both high precision and recall across tumor classes, even when the dataset is imbalanced.

### 5.4. Class-Wise Performance Metrics and Detailed Analysis

For a comprehensive understanding of the model’s performance, besides overall accuracy, we utilized four critical metrics: precision, recall, F1-score, and specificity. All of these metrics allow a thorough assessment of how well the model performs in identifying each of the breast cancer subtypes present in the BreaKHis dataset. Among these metrics, precision reflects the proportion of samples that were correctly identified as positive among all those predicted as positive. Precision is important in medical diagnosis, where a high precision score corresponds to a low false positive rate, which can minimize unnecessary treatments or invasive procedures. Similarly, recall measures the ability of the model to identify all the true positives within each sample in a given class. A high recall score ensures that all actual cancer cases in a clinical setting are diagnosed. In an imbalanced dataset, the F1-score plays a vital role in providing a measure of the model’s completeness in identifying positive cases. Since the F1-score represents the harmonic mean of precision and recall, it provides a score reflecting both the accuracy of positive predictions and the model’s completeness in identifying positive cases. In the end, specificity reflects the ratio of correctly identified negative cases, thereby ensuring that the model does not raise false alarms while identifying non-cancerous or different subtype cases.

**Figure 6:**
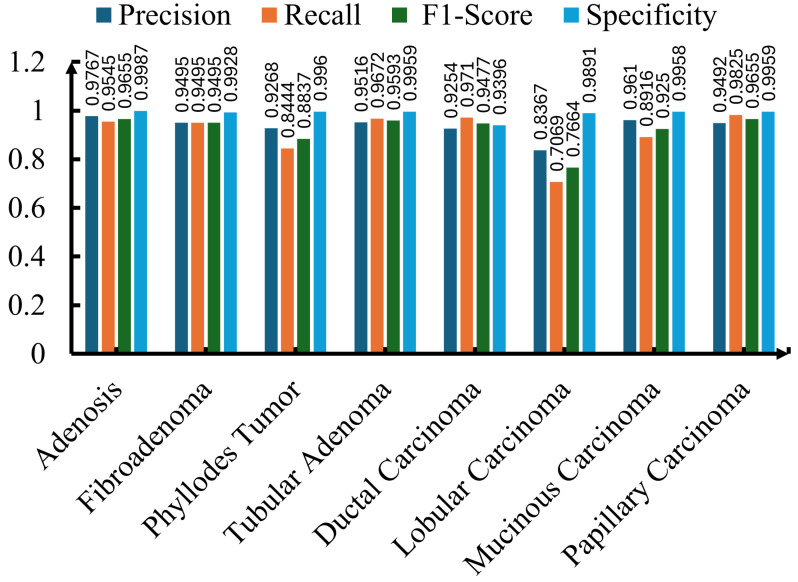
Precision, recall, F1-score, and specificity for each of the eight breast cancer classes using the ResNet-50 model.

Although all three ResNet models achieved impressive performance across all four metrics, ResNet-50 exhibited superior performance, and its evaluation is shown in Figure 6. Precision for each of the seven breast cancer classes was more than 93%, except for lobular carcinoma. For the same class (lobular carcinoma), the F1-score and recall were under 78%, likely due to the subtle visual differences with other subtypes or due to fewer training samples. Except for lobular carcinoma, the model exhibited superior performance for other subtypes. For instance, adenosis achieved the highest scores in each category, including a precision of 0.9767, a recall of 0.9545, and an F1-score of 0.9655, showing the trained model’s exceptional ability in identifying adenosis with minimal misclassifications. Similar to adenosis, tubular adenoma, fibroadenoma, and papillary carcinoma also showed superior scores in precision, recall, and F1-score. The model achieved impressive scores of 0.9710 and 0.9477 in recall and F1-score, respectively, for ductal carcinoma, one of the most prevalent and clinically significant subtypes of malignant tumors. This shows that the trained model can play a pivotal role in detecting this critical class with minimal false negatives, as shown in Figure 6. Besides precision, recall, and F1-score, the model consistently achieved high specificity, with each subtype scoring more than 0.92. In some cases, such as adenosis, tubular adenoma, and papillary carcinoma, the specificity score approached near-perfect values. This shows that the trained ResNet-50 model has the ability to minimize false positive predictions, which is an essential characteristic in clinical applications.

To measure the impact and effectiveness of the proposed ResNet model, it is crucial to compare it with existing published works that also utilized the BreaKHis dataset and applied multi-class classification on breast tumor subtypes. This comparison is presented in Table 6, where the proposed technique is shown to achieve superior accuracy compared to previously reported methods that achieved accuracies ranging from 73.68% to 91.3%. In these methods, various techniques such as traditional CNNs, Inception V3, and attention-based networks like ECSAnet were employed. For most of the methods shown in Table 6, the datasets were split in an 80:20 ratio for the training and test sets. In some cases, they were split into training, test, and validation sets. For uniformity, the validation and test divisions are combined into one category. This comparison clearly shows the robustness and practical potential of the proposed model in automating breast cancer subtype classification for clinical pathology.

### 5.5. Impact of Class Imbalance Mitigation Strategies

**Figure 7:**
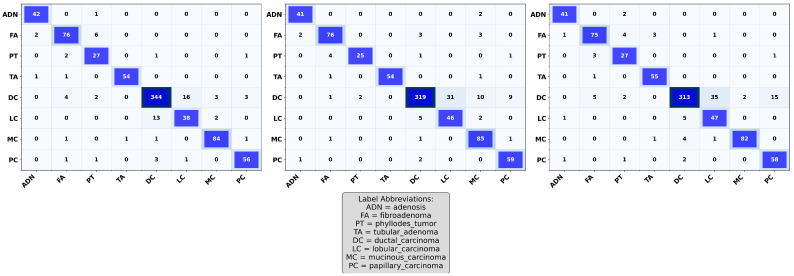
Comparison of confusion matrices for ResNet-50 using baseline (**left**), balanced oversampling (**middle**), and focal loss (**right**) techniques.

As shown in Figure 6, among all subtypes, ResNet-50 performed less satisfactorily in classifying lobular carcinoma. This is due to the class imbalance in the dataset, where the number of sample histopathological images varies significantly across breast cancer subtypes. Therefore, class-balanced oversampling and the focal loss technique were applied to address this severe class imbalance in the BreaKHis dataset. These approaches were evaluated along with the baseline model trained with random shuffling utilized in this work. Utilizing focal loss and balanced oversampling improved ResNet-50’s ability to identify lobular and papillary carcinoma compared to random shuffling, as shown in Figure 7. However, the performance of focal loss and balanced oversampling either deteriorated or was similar in other classes. This was especially evident in ductal carcinoma. Hence, the two data-balancing techniques did not yield substantial improvements over the baseline model.

### 5.6. Class-Wise Performance Across Magnifications

**Figure 8:**
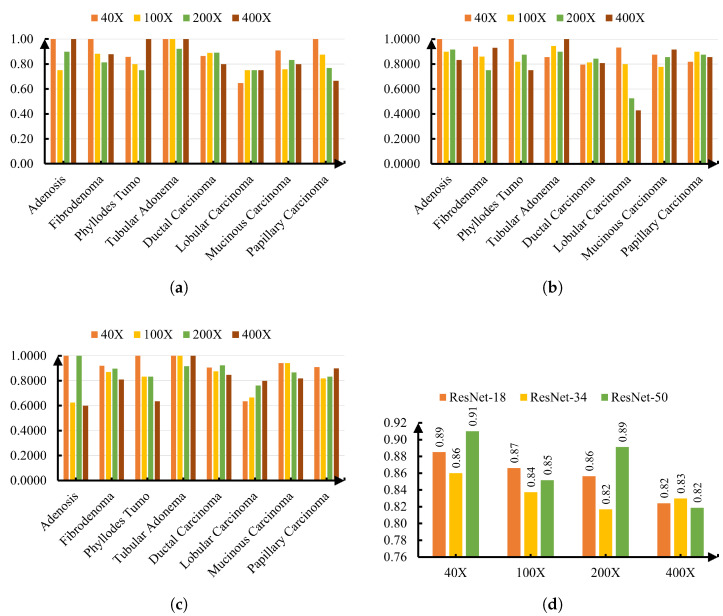
Performance across magnifications for different ResNet variants. (**a**) Precision per class for ResNet-18 across magnifications. (**b**) Precision per class for ResNet-34 across magnifications. (**c**) Precision per class for ResNet-50 across magnifications. (**d**) Overall accuracy across magnifications for all ResNet variants.

Thus far, the accuracy of each ResNet model and its performance for each of the classes have been measured. However, to evaluate the robustness of the proposed models, it is also important to report the class-wise performance for each magnification level (40×, 100×, 200×, and 400×) rather than collapsing the results into a single aggregated figure. This shows the effect of magnification on the accuracy of each ResNet model and its performance in classifying histopathological images. A comparison of the accuracies of ResNet-18, -34, and -50 for various magnification levels (40×, 100×, 200×, and 400×) is shown in Figure 8d. ResNet-50 performed better than ResNet-18 and ResNet-34 for 40X magnification and 200X magnification. Meanwhile, ResNet-34 and ResNet-18 outperformed their counterparts for 100X magnification and 400X magnification, respectively.

In addition to accuracy, precision was evaluated to provide a more detailed view of model performance for each tumor subtype across different magnifications. High precision is particularly important in medical diagnosis, as it reflects the likelihood that a positive classification truly corresponds to the target condition, thereby reducing the risk of unnecessary treatments. All ResNet models selected in this work showed superior precision in classifying tubular adenoma at all four magnification levels. Meanwhile, lobular carcinoma exhibited the most variability, with precision dropping below 0.70 in multiple cases, particularly for ResNet-34 at 200× (0.5263) and 400× (0.4286), suggesting greater sensitivity to magnification changes and possible feature overlap with other subtypes. On the other hand, for phyllodes tumor, the performance of ResNet-18 and ResNet-50 was much more consistent across different magnifications than ResNet-34. These findings highlight the effect of magnification and network depth on classification performance for certain types of cancer and accuracy.

### 5.7. Visualization of Model Predictions

**Figure 9:**
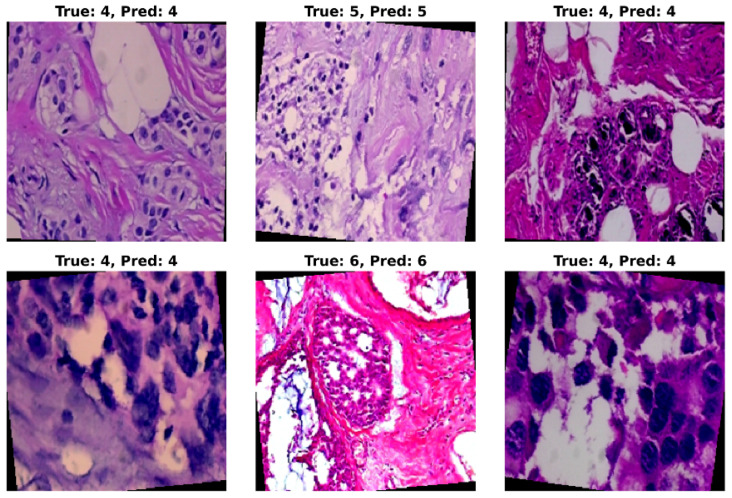
Visualization of ResNet-50 model predictions on randomly selected histopathological images from the BreaKHis test set. Each image displays the corresponding ground-truth class (**True**) and the predicted class (**Pred**) assigned by the model. The visualization highlights the model’s strong ability to correctly classify most tumor subtypes at varying magnifications, while also revealing occasional misclassifications in subtypes with subtle morphological differences.

Through quantitative metrics, we were able to showcase the effectiveness of the ResNet models in classifying breast cancer into eight subtypes. To complement the numerical results, a visualization of the trained model’s prediction ability provides valuable insights into classification performance. For this purpose, randomly selected test set images were applied as input to the model, and the predicted classification was then compared with the original class label. A grid of six histopathological images from the BreaKHis test set, representing various breast cancer subtypes and magnification levels, was provided as input to the trained ResNet-50 model. The model’s predictions (Pred), alongside the true class labels (True), are shown in Figure 9. In Figure 9, True class 4 represents ductal carcinoma, class 5 represents lobular carcinoma, and class 6 corresponds to mucinous carcinoma. In this figure, most of the predictions on the test samples are shown to be correct. However, on some occasions, misclassification may occur when the subtypes exhibit subtle morphological similarities.

### 5.8. Model Interpretability and Explanation

**Figure 10:**
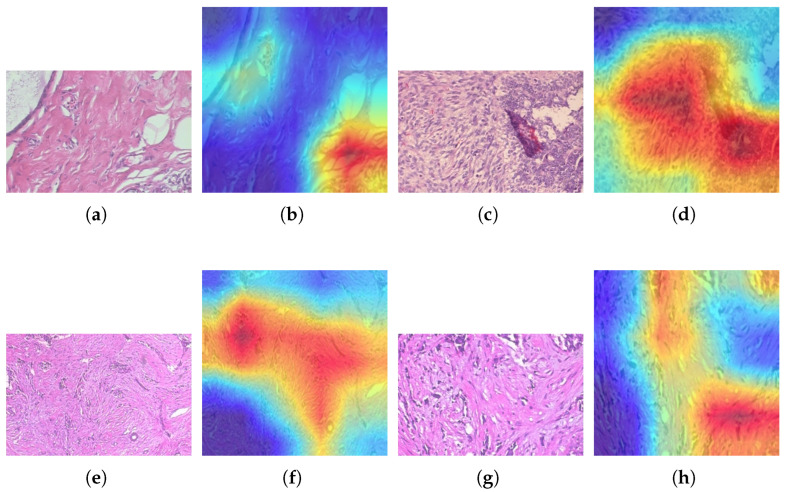
Grad-CAM visualizations: (**a**) original benign adenosis sample, (**b**) Grad-CAM of benign adenosis, (**c**) original benign phyllodes tumor sample, (**d**) Grad-CAM of benign phyllodes tumor sample, (**e**) original ductal carcinoma sample, (**f**) Grad-CAM of ductal carcinoma misclassified as lobular carcinoma, (**g**) original ductal carcinoma sample, (**h**) Grad-CAM of ductal carcinoma misclassified as lobular carcinoma.

Visualizing the performance of the ResNet models in classifying breast cancer types is essential; however, the models must be able to highlight the histopathology images when they make correct or incorrect predictions. This can be achieved by using Gradient-weighted Class Activation Mapping (Grad-CAM). Among the ResNet models, ResNet-50 achieved the highest accuracy. A qualitative visualization of its performance using Grad-CAM on various histopathology images across various classes from the BreaKHis dataset is shown in Figure 10. Warmer colors indicate a more substantial positive contribution to the selected class. On the other hand, cooler or lighter colors indicate a small or no contribution. Figure 10a shows an original adenosis sample at a magnification of 100X. The ResNet-50 model correctly identified this sample. Figure 10b shows the Grad-CAM of the same histopathology image, where heat concentrates over crowded glandular/acinar units within the preserved lobular architecture. Similarly, another correctly predicted sample from the phyllodes tumor category at 100X magnification and its Grad-CAM representation are shown in Figure 10c and Figure 10d, respectively. Besides correct predictions, it is vital to see what Grad-CAM highlights when the ResNet model makes incorrect predictions to better understand the reason behind it. Hence, in Figure 10e,g, the original ductal carcinoma sample is selected at a different magnification, which the ResNet-50 classified incorrectly as lobular carcinoma. This visualization validates both successful classifications and explains the specific failure modes during misclassification.

## 6. Discussion

This work discusses the potential of deep learning models, such as the ResNet architecture, to achieve the challenging task of multi-class classification of breast cancer subtypes from histopathological images. Among ResNet-18, ResNet-34, and ResNet-50, the latter outperformed the others across all metrics and parameters, including accuracy, AUC, ROC, precision, recall, F1-score, and specificity. This superior performance can largely be attributed to the deeper architecture of ResNet-50, specifically its bottleneck residual blocks, which enable the extraction of complex, fine-grained histopathological features essential for differentiating among visually similar subtypes. The model performed much better in classifying subtypes such as ductal carcinoma, adenosis, fibroadenoma, and tubular adenoma. Even though the model performed better than other published works in classifying lobular carcinoma and mucinous carcinoma, its performance for these subtypes was still not as strong as for others, likely due to the subtle morphological differences among these subtypes and their under-representation within the dataset. Hence, due to these observations, further research is necessary, where more advanced data augmentation strategies, subtype-specific learning approaches, and more balanced datasets will be used to improve the classification of challenging subtypes.

### 6.1. Practical Deployment Scenarios

A highly accurate, trained AI model is insufficient without an actual plan for evaluating the model’s applicability in real-world clinical workflows. The proposed model can be deployed in two different scenarios, as outlined below.

#### 6.1.1. Workstation-Integrated Microscope

In this approach, a workstation uses a histological microscope to capture images, deploys the trained ResNet model in the same environment, and promptly classifies the breast cancer type [37]. In the preprocessing steps, the captured image is resized to 224 × 224 pixels, and after normalization, it is fed as input to the ResNet model, which classifies the histopathological image into one of the eight categories. In this scenario, pathologists can utilize and interact with an AI model for diagnosis, fostering a “dialogue-mode” workflow that combines human expertise with machine precision.

#### 6.1.2. Remote Processing Workflow

In some scenarios, the trained AI model needs more computational resources, making it impossible to deploy using the workstation-integrated microscope setup. In such cases, histopathological slides are digitized and transferred to a centralized server for analysis. Typically, in this type of setup, images undergo manual or semi-automated preprocessing, including format conversion and spatial resolution adjustments. After preprocessing, the image is applied to the trained model, which classifies it into one of the categories. Although this approach requires access to high computational resources, it suffers from latency issues due to data transfer and preprocessing overhead.

### 6.2. Future Work

In a recent work conducted using an ensemble of Swin Transformers, on the BreaKHis dataset, BreaST-Net achieved a higher test accuracy of 96% across eight subtypes (at 40× magnification) [38]. However, some of the metrics used in the model were performance-related. Similarly, Joseph et al. used a hybrid method combining a handcrafted feature extraction technique and a deep neural network (DNN) classifier, achieving an accuracy of 97.89% in multi-class classification of breast cancer subtypes on the same dataset [39]. Likewise, Chikkala et al. proposed a novel bidirectional recurrent neural network (BRNN) framework that integrates a ResNet-50-based transfer learning backbone, Gated Recurrent Units (GRUs), residual collaborative branches, and a feature fusion module for multi-class classification on the BreaKHis dataset, achieving an accuracy of 97.25% [40]. Sharma et al. used a VGG16 pretrained network with a linear SVM classifier on the BreaKHis dataset, achieving an accuracy slightly better than that of the ResNet-50 model proposed in this work [41]. These recent publications highlight that by incorporating advanced architectures, such as transformers or recurrent neural networks, or by using hybrid learning approaches that integrate handcrafted features, multi-classification of breast cancer can be achieved. While these models achieved slightly higher accuracies than the ResNet-50 model presented in this study, they often require more complex architectures or additional training resources. Additionally, the integration of explainable AI (XAI) methods could also improve the interpretability of model decisions, fostering greater trust in clinical applications. In future work, we will incorporate these advanced methods into the current framework, along with additional techniques such as ensemble learning and domain adaptation, to improve accuracy and generalizability. Recently, Attention GhostUNet++ achieved better contextual understanding and feature refinement on liver CT images, with efficient computational resource requirements [42]. Hence, comparing the ResNet models presented in this work with Attention GhostUNet++ is pertinent. Moreover, in future work, we will also investigate the effect of using native image resolutions or a patch-based strategy, as discussed in [43], rather than the uniform resizing of 224 × 224 employed in this work. Overall, this study reinforces the capability of deep learning for multi-class classification of histopathological images and lays a strong foundation for continued research in this domain.

## 7. Conclusions

In this study, we developed and rigorously evaluated a deep learning framework for multi-class classification of eight breast cancer subtypes on the BreaKHis dataset using ResNet-18, ResNet-34, and ResNet-50. Across five random seeds, ResNet-50 delivered the strongest and most stable performance (mean test accuracy was equal to 92.42% ± 1.30; 95% CI: 91.13–93.72), with high discrimination on threshold-free metrics (overall AU-ROC up to 99.57) and robust class-wise precision–recall behavior (PR-AUC ≥ 0.91 for all classes, with several ≥ 0.99). Confusion-matrix analyses showed consistently correct recognition of adenosis, fibroadenoma, tubular adenoma, and ductal carcinoma, while the model most often confused lobular with mucinous carcinoma and, to a lesser extent, phyllodes tumor with fibroadenoma, patterns aligned with known morphological similarity. Grad-CAM visualizations indicated that the network attends to histologically meaningful regions in both correct and error cases, providing qualitative interpretability that can support pathologist review. Beyond histopathology, the proposed solutions are potentially suitable for multi-class breast tumor classification based on X-ray mammography, ultrasonography, and magnetic resonance imaging, provided appropriate modality-specific preprocessing and retraining. Future work will investigate cross-modality transfer learning and domain adaptation to validate this potential on external, multi-institutional cohorts.

Despite these strengths, limitations remain. Performance is partially constrained by class imbalance and dataset size, and our evaluation is limited to a single public cohort; external, multi-institutional validation is necessary to establish generalizability. Additionally, because the task presented in this work is image-level classification, not segmentation, our assessment appropriately focused on classification metrics (accuracy, AUC-ROC, PR-AUC, precision, recall, F1-score, and specificity) rather than surface-distance measures.

Future work will prioritize (i) patient-level or leave-one-patient-out splitting to further mitigate potential leakage, (ii) stain normalization and domain adaptation to reduce site-specific bias, (iii) ensemble and transformer/hybrid backbones for harder subtypes, and (iv) expanded interpretability studies and prospective, pathologist-in-the-loop evaluation. Taken together, our results establish a strong, reproducible baseline for subtype-level histopathology classification and outline a clear path toward clinically reliable, AI-assisted decision support.

## Figures and Tables

**Table 1:** Four magnification levels in BreaKHis, categorized into benign and malignant tumor subtypes.

Magnification Level	Description	Application
40X	Low-resolution overview of tissue structure	Identifying overall morphology
100X	Balanced detail of cell structure and tissue morphology	Intermediate analysis
200X	Detailed examination of cellular organization	Feature extraction for AI models
400X	High-resolution visualization of individual cell structures	Fine-grained classification

**Table 2:** Tumor subtypes in the BreaKHis dataset. The dataset includes both benign and malignant tumors, each further categorized into four distinct subtypes.

Benign Tumors	Malignant Tumors
**Adenosis:** Non-cancerous overgrowth of glands within the lobules	**Ductal Carcinoma:** The most common malignant tumor, originating in the milk ducts
**Fibroadenoma:** Common benign tumor composed of fibrous and glandular tissues	**Lobular Carcinoma:** Cancer that begins in the lobules and tends to spread diffusely
**Phyllodes Tumor:** Rare fibroepithelial tumor with potential to recur	**Mucinous Carcinoma:** Malignant tumor characterized by mucin production
**Tubular Adenoma:** Well-circumscribed benign tumor of tightly packed tubules	**Papillary Carcinoma:** Malignant tumor with papillary structural patterns

Tumor descriptions have been adapted for clarity.

**Table 3:** Comparison of ResNet architectures utilized in this study.

ResNet Model	Depth	Residual Block Type	Parameters (Millions)
ResNet-18	18 layers	Basic Block	11.7
ResNet-34	34 layers	Basic Block	21.8
ResNet-50	50 layers	Bottleneck Block	25.6

**Table 4:** Summary of ResNet architectures for breast cancer subtype classification.

Metric	ResNet-18	ResNet-34	ResNet-50
Total Parameters	11,180,616	21,288,776	23,524,424
Model Size (MB)	42.65	81.21	89.74
GFLOPs	1.824 G	3.678 G	4.132 G
Inference Time (ms)	3.67 ± 0.07	5.15 ± 0.14	5.54 ± 0.15
Input Resolution	224 × 224	224 × 224	224 × 224
Output Classes	8	8	8
Backbone	ResNet-18	ResNet-34	ResNet-50
Pretrained Weights	ImageNet	ImageNet	ImageNet

**Table 5:** Performance summary across 5 random seeds with mean accuracy and 95% confidence intervals.

Model	Test Accuracies (Seeds)	Mean Accuracy (%)	95% CI (%)
ResNet-18	[85.86, 90.15, 89.89, 87.88, 89.89]	88.74 ± 2.30	(86.44, 91.04)
ResNet-34	[86.74, 88.76, 86.49, 90.66, 89.65]	88.46 ± 2.25	(86.21, 90.71)
ResNet-50	[94.19, 92.42, 92.17, 91.67, 91.67]	92.42 ± 1.30	(91.13, 93.72)

**Table 6:** Comparison of classification performance with existing methods.

Work	Method	Dataset Split (Train:Test)	Classification Type	Accuracy
[33]	CNN	70:30	8 Class	88.23%
[34]	Inception V3 CNN	80:20	8 Class	88.16%
[35]	CNN	90:10	8 Class	73.68%
[36]	ECSAnet	70:30	8 Class	91.3%
Proposed	ResNet-18	80:20	8 Class	88.74% ± 2.30%
Proposed	ResNet-34	80:20	8 Class	88.46% ± 2.25%
Proposed	ResNet-50	80:20	8 Class	92.42% ± 0.98%

## Data Availability

The BreaKHis dataset used in this study is publicly available at https://www.kaggle.com/datasets/ambarish/breakhis or via the original website at http://web.inf.ufpr.br/vri/databases/breast-cancer-histopathological-database-breakhis/, accessed on 30 October 2024.
